# The Probiotic Combination of *Bifidobacterium longum* subsp. *infantis* CECT 7210 and *Bifidobacterium animalis* subsp. *lactis* BPL6 Reduces Pathogen Loads and Improves Gut Health of Weaned Piglets Orally Challenged with *Salmonella* Typhimurium

**DOI:** 10.3389/fmicb.2017.01570

**Published:** 2017-08-15

**Authors:** Emili Barba-Vidal, Lorena Castillejos, Victor F. B. Roll, Gloria Cifuentes-Orjuela, José A. Moreno Muñoz, Susana M. Martín-Orúe

**Affiliations:** ^1^Animal Nutrition and Welfare Service, Departament de Ciència Animal i dels Aliments, Universitat Autònoma de Barcelona Bellaterra, Spain; ^2^Department of Animal Science, Faculty of Agronomy Eliseu Maciel, Federal University of Pelotas Pelotas, Brazil; ^3^Investigación Básica, Laboratorios Ordesa S.L. Barcelona, Spain

**Keywords:** multi-strain probiotic, *Bifidobacterium* sp., pig model, *Salmonella* Typhimurium, microbiota, diarrhea, infant model

## Abstract

Probiotics have been demonstrated to be useful to enhance gut health and prevent gastrointestinal infections in humans. Additionally, some multi-strain probiotic combinations have been suggested to have greater efficacy than single strains. The objective of this study is to demonstrate the potential of a combination of the probiotic strains: *Bifidobacterium longum* subsp. *infantis CECT 7210* (brand name *B. infantis* IM1^®^) and *B. animalis* subsp. *lactis* BPL6 to enhance gut health and to ameliorate the outcome of a *Salmonella* challenge using a weaning piglet model. Seventy-two 28-day-old weanling piglets, 7.7 (±0.28) kg of body-weight, were distributed in a 2 × 2 factorial design; treated or not with the probiotic combination and challenged or not with the pathogen. Animals were orally challenged after an adaptation period (Day 8) with a single dose (5 × 10^8^ cfu) of *Salmonella* Typhimurium. One animal per pen was euthanized on Day 12 (Day 4 post-inoculation [PI]) and Day 16 (Day 8 PI). All parameters responded to the challenge and 4 deaths were registered, indicating a severe but self-limiting challenge. Improvements registered in the challenged animals due to the probiotic were: increased voluntary feed-intake (*P* probiotic × challenge = 0.078), reduced fecal excretion of *Salmonella* (*P* = 0.028 at Day 1 PI and *P* < 0.10 at Days 3 and 5 PI), decreased rectal temperature (*P* probiotic × day = 0.048) and improvements in the villous:crypt ratio (*P* probiotic × challenge < 0.001). Moreover, general probiotic benefits were observed in both challenged and non-challenged groups: decreased diarrhea scores of the PI period (*P* = 0.014), improved fermentation profiles on Day 8 PI (increased ileal acetic acid [*P* = 0.008] and a tendency to lower colonic ammonia concentrations [*P* = 0.078]), stimulation of intestinal immune response by increasing villous intraepithelial lymphocytes (*P* = 0.015 on Day 8 PI) and an improved villous:crypt ratio (*P* = 0.011). In conclusion, the multi-strain probiotic had a positive effect on reducing pathogen loads and alleviating animals in a *Salmonella* challenge. In addition, enhanced gut health and immunity was recorded in all animals receiving the probiotic, indicating an improvement in the post-weaning outcome.

## Introduction

*Salmonella* spp. enteric infections are among the most common diarrhea-associated causes of morbidity and mortality (CDC, 2013), especially in children up to 5 years of age (Lanata et al., 2013). In fact, newborn babies are considered to be especially vulnerable, as their immune system is still not fully developed and may be prone to infections by opportunistic pathogens (Lanata et al., 2013; Thanabalasuriar and Kubes, 2014). Altogether, *Salmonella* infections are estimated to be responsible for up to 155,000 deaths, when considering the global population (Majowicz et al., 2010), and over 100,000 human clinical cases are reported each year only in the EU, with an estimated overall economic burden of human salmonellosis of 3 billion euros a year (EFSA, 2013).

Probiotics and their metabolites have been suggested to play an important role in the formation or establishment of well-balanced, indigenous, intestinal microbiota in new-borne infants and adults (Gill, 2003; Salazar et al., 2009). Furthermore, the administration of probiotic microorganisms in milk formulas has well-documented benefits, including improvements in infections, diarrhea, allergic disorders, various gut pathogens and inflammatory diseases in children (Bin-Nun et al., 2005; Collado et al., 2007c; Minocha, 2009). For instance, remarkable beneficial effects against *Salmonella* have been documented by the *Bifidobacterium* spp. genus, with well-documented research *in vitro* (Liévin et al., 2000; Tanner et al., 2016) and *in vivo* using animal models (Shu et al., 2000; Silva et al., 2004; Zacarías et al., 2014). In particular, one of the strains conforming the probiotic combination tested in this study, *Bifidobacterium longum* subsp. *infantis* CECT 7210 (*B. infantis* IM1^®^), has demonstrated a reduction in ETEC K88 colonization and *Salmonella* fecal shedding in an *in vivo* model with weanling pigs (Barba-Vidal et al., 2017) and protective effects against a rotavirus infection *in vitro* and in a murine model (Moreno Muñoz et al., 2011).

Multi-strain and multi-species probiotic combinations have been suggested to have greater efficacy than single strains, as complementary or even synergistic effects can be achieved in the host when given together in comparison to giving them separately (Timmerman et al., 2004; Collado et al., 2007b; Chapman et al., 2011). Nevertheless, each specific probiotic combination should be tested, and further research efforts are needed. In this study, we hypothesize that combining the probiotic strain of *B. longum* subsp. *infantis* CECT 7210 with *B. animalis* subsp. *lactis* BPL6 could improve gut health. The objective of this work was, therefore, to demonstrate the potential of this probiotic combination to enhance gut health in human early-life stages and to ameliorate the outcome of a *Salmonella* challenge using a weaning piglet model.

## Materials and Methods

The experiment was performed at the Experimental Unit of the Universitat Autònoma de Barcelona (UAB) and received prior approval (Permit No. CEAAH1619) from the Animal and Human Experimental Ethical Committee of this Institution. The treatment, management, housing, husbandry and slaughtering conditions conformed to European Union Guidelines (European Commission, 2010), and all efforts were made to minimize animal suffering.

### Animals and Housing

The trial was conducted as a Level 2 High-Risk Biosecurity Procedure, with appropriate training of the personnel involved. A total of 72 male piglets (Large White × Landrace) from a high-sanitary-status farm and from mothers serologically negative to *Salmonella* were used. Animals were weaned at 28 (±3) days of age, 7.7 (±0.28) kg body-weight (BW) on average, and were transported to the UAB facilities, where they were placed in three rooms of eight pens each (24 pens, three animals per pen), taking initial BW into account for a similar average BW within pens. The pens were allocated to four treatment groups following an unbalanced 2 × 2 factorial arrangement (factors being probiotic and pathogen challenge), with eight replicates per treatment for the challenged animals and four replicates for the non-challenged group. The treatments were, therefore: (1) no challenge + no probiotic (NN); (2) no challenge + probiotic (NP); (3) challenged + no probiotic (CN) and (4) challenged + probiotic (CP). Two rooms were challenged with pathogens and one was left unchallenged. In each room, probiotic treatment was distributed among four pens on one side of the room, and the four control pens were on the other side of the room, separated by a corridor in between.

Pigs were maintained under a 14:30 h light/9:30 h dark lighting regimen. Each pen (2 m^2^) had a feeder and a water nipple to provide feed and water for *ad libitum* consumption. The weaning rooms were equipped with automatic heating, forced ventilation and an individual heat-light per pen. The experiment was conducted during the spring season (April), with an average room temperature of 26°C (±4°C). The experimental treatments were distributed evenly among the three rooms.

### Experimental Products and Diets

The probiotic treatment was supplied by Ordesa S.L., and it consisted of a daily dosage (10^9^ colony-forming units [cfu]) of a combination of *B. longum* subsp. *infantis* CECT 7210 (*B. infantis* IM1^®^) and *B. animalis* subsp. *lactis* BPL6 (*B. lactis* BPL6), supplemented in a 2 mL solution. The control group received, as a placebo, the same amount of carrier. During the experimental period, pigs received the treatment orally and individually, in a daily pattern using disposable 2 mL syringes without a needle. The probiotic tested was a single batch of lyophilized bacteria, which was re-suspended and administered every day, in less than 1 h time period. Viability of probiotic administered was verified by plating bacteria shortly after re-suspending the probiotic, and 1 and 2 h later stored at room temperature. Serial dilutions of the suspension were performed in Man Rogosa Sharpe (MRS) broth (Oxoid; Madrid, Spain) + 0.25% cysteine (Sigma–Aldrich; Madrid, Spain), plated in MRS-C agar (Oxoid; Madrid, Spain), incubated at 37°C in anaerobic conditions for 48 h and manually counted. Colony forming units were always maintained in a logarithm scale of 10^9^ cfu/g.

A pre-starter diet without additives (**Table 1**) was formulated to satisfy the nutrient requirement standards for pigs of this age (NRC, 2012) and was given in a mash form.

**Table 1:** Ingredient composition and nutrient analysis of the experimental diets as-fed basis, g/kg.

**Ingredients**	
Maize	280.7
Wheat	170.0
Barley	150.0
Extruded soybean	122.4
Sweet whey-powder	100.0
Fishmeal LT	50.0
Soybean meal 44	50.0
Whey-powder 50% fat	30.3
Mono-calcium phosphate	21.3
Calcium carbonate	8.2
L-Lysine HCL	4.5
Vitamin-Mineral Premix^a^	4.0
Sodium chloride	3.0
DL-Methionine 99	2.4
L-Threonine	2.3
L-Tryptophan	0.9
**Chemical composition**	
DM	903.2
Ash	74.1
Crude fat	64.5
Crude protein	189.3
Neutral detergent fiber	111.6
Acid-detergent fiber	35.1

*^a^Provided per kilogram of complete diet: 10,200 IU vitamin A, 2,100 IU vitamin D_*3*_. 39.9 mg vitamin E, 3 mg vitamin K_*3*_, 2 mg vitamin B_*1*_, 2.3 mg vitamin B_*2*_, 3 mg vitamin B_*6*_, 0.025 mg vitamin B_*12*_, 20 mg calcium pantothenate, 60 mg nicotinic acid, 0.1 mg biotin, 0.5 mg folic acid, 150 mg Fe, 156 mg Cu, 0.5 mg Co, 120 mg Zn, 49.8 mg Mn, 2 mg I, 0.3 mg Se.*

### Bacterial Strain

The bacterial strain used in the present study was a *Salmonella* Typhimurium var. Monophasic (formula: 4,5,12:i:-, resistance profile: ACSSuT-Ge, Fagotype: U302) that was isolated from a salmonellosis outbreak (mainly enteric and with sporadic septicemia) of fattening pigs in Spain, and was provided by the Infectious Diseases Laboratory (Ref. 301/99) of the UAB. The oral inoculum was prepared by 24 h incubation at 37°C in buffered peptone water (BPW) (Oxoid; Hampshire, United Kingdom) and diluted (1:20) with sterile phosphate buffered saline (PBS) (Sigma–Aldrich; Madrid, Spain) to reach a final concentration of 2.5 × 10^8^ cfu/mL.

### Experimental Procedure

The duration of the study was 16 days, in which performance and clinical data were evaluated. After 1 week of adaptation to the diets (Day 8), a single 2 mL dose (5 × 10^8^ cfu) of *Salmonella* Typhimurium was administered to the challenged animals by oral gavage and a single 2 mL dose of sterile BPW to the non-challenged animals (challenge control group).

Body weight was recorded on Days 1, 8, 12 and 16, while feed consumption was recorded on Days 1 and 7, and on a daily basis of the post-inoculation (PI) period (Days 8–16). The average daily gain (ADG), average daily feed intake (ADFI) and gain:feed ratio (G:F) were calculated by pen. Animals were checked daily for clinical signs to evaluate their status (i.e., dehydration, apathy and fecal score) after the *Salmonella* challenge, always by the same person. The fecal score was measured using a scale: 1 = solid and cloddy, 2 = soft with shape, 3 = very soft or viscous liquid, and 4 = watery or with blood. Rectal temperature was assessed with a digital thermometer (Thermoval Rapid, Hartmann; Spain) on Days 9 and 10 (1 and 2 PI). The mortality rate was also recorded, and no antibiotic treatment was administered to any of the animals of the experiment.

For microbiological analysis, on Day 1 fecal samples were taken aseptically from 24 animals that were randomly selected from the total before distribution. Samples were taken after spontaneous defecation associated with the manipulation of the animal or by digital stimulation. On Days 8, 9, 11 and 15 (Days 0, 1, 3 and 7 PI), fecal samples were taken from the animal with the highest initial BW of each pen (*N* = 24).

On Days 4 and 8 PI (Experimental Days 12 and 16, respectively), one pig per pen was euthanized. On Day 4 PI, the animal selected was the one with the intermediate initial BW, while on Day 8 PI the heaviest was selected.

Animals were euthanized and sequentially sampled during the morning (between 09:00 and 12:00 h). Prior to euthanasia, a 10 mL sample of blood was obtained by venipuncture of the cranial vena cava using 10 mL tubes without anticoagulant (Aquisel; Madrid, Spain). Immediately after blood sampling, selected piglets received an intravenous lethal injection of sodium pentobarbital (200 mg/kg body weight) (Dolethal; Vetoquinol S.A.; Madrid, Spain). Once dead, animals were bled, the abdomen was immediately opened and the whole gastrointestinal tract was excised.

Digesta (approximately 50 mL) from the ileum and proximal colon (considered to be 0.75 m from the ileocecal junction) was collected and homogenized. The pH of the digesta was determined immediately after homogenization of the samples with a pH-meter calibrated on each day of use (Crison 52–32 electrode, Net Interlab; Barcelona, Spain). Without delay, contents collected were sub-sampled and kept on ice until analysis or were stored. Colonic samples (1 g) were plated for *Salmonella* quantification. To determine the presence of the probiotic in the gut, 2 g of digesta were sampled (only on Day 8 PI) and bacterial isolation was performed before storing them at -80°C with GenIUL commercial protocol (Terrassa, Spain). Briefly, 1 g of colonic content sample was weighed in a 15 mL falcon and diluted 1:10 with enriched MRS broth (Oxoid; Madrid, Spain) + 0.25% cysteine (Sigma–Aldrich; Madrid, Spain) + 2% Tween 80 (Sigma–Aldrich; Madrid, Spain). Ten glass spheres (5 mm diameter) were added to the tube and vortex (1 min) to homogenize the suspension. Two-hundred and fifty microliter of the sample suspension were transferred to an eppendorf with 250 μL of enriched MRS broth. Three centrifugation (13,000 × *g* for 5 min at 4°C) and re-suspension (500 μL of enriched MRS broth) steps were performed, and finally the bacterial pellet was re-suspended in 200 μL of sterile PBS and stored at -80°C for DNA extraction and quantification via quantitative polymerase chain reaction (qPCR). A set of ileal and colonic digesta samples were preserved in a H_2_SO_4_ solution (3 mL of digesta plus 3 mL of 0.2 N H_2_SO_4_) for ammonia (NH_3_) determination, and were kept frozen at -20°C. An additional ileal and colonic sample set (approximately 20 g) was also frozen (-20°C) until analyzed for short-chain fatty acids (SCFA) and lactic acid.

For the histological study, 3 cm sections of the ileum were removed, opened longitudinally, washed thoroughly with sterile PBS and fixed by immersion in a 4% formaldehyde solution (Carlo-Erba Reagents; Sabadell, Spain).

Blood samples were centrifuged (3,000 × *g* for 15 min at 4°C) after 4 h refrigeration, and the serum obtained was divided into different aliquots and stored at -20°C to evaluate immune response.

### Analytical Procedures

Chemical analyses of the diets including DM, ash, crude protein and diethyl ether extract, were performed according to the Association of Official Agricultural Chemists standard procedures (AOAC International, 1995). Neutral detergent fiber and ADF were determined according to the method of Van Soest et al. (1991).

For probiotic detection, DNA was extracted with a commercial kit following the manufacturer’s instructions (v-DNA reagent, Doc. Code 450000112.GenIUL; Terrassa, Spain). Briefly, samples were suspended in 1 mL of v-DNA buffer and centrifuged (13,000 × *g* for 5 min at 4°C). An incubation of the bacterial pellet (90°C, 10 min) with 200 μL of v-DNA reagent was performed in a shaking incubator and, finally, DNA was suspended in 600 μL of v-DNA buffer. The *GenIUL Bifidobacterium* spp. qPCR kit was used for probiotic quantification (reference: 4900021000, GenIUL; Terrassa, Spain). The kit provided a *Bifidobacterium longum* CECT 4551 DNA standard for the construction of standard curves (from 2 × 10^6^ to 20 DNA copies per PCR reaction). Each reaction included 4 μl of a 5× HOT FIREPol qPCR Master mix including qPCR assay primers designed for the 16s RNA gene, 5 μl of diluted (1/10) DNA samples and 11 μl of RNAse free water. Reaction conditions for amplification of DNA were 95°C for 15 min and 45 cycles of 95°C, 15 s for denaturation, 54°C, 30 s for annealing and 72°C, 45 s for extension and fluorescent detection. To determine the specificity of the amplification, an analysis of the product-melting curve was performed after the last cycle of each amplification. The minimum level of detection of the method, considering the amount of DNA included in each reaction, was established at 6.3 × 10^3^ 16 S ribosomal RNA gene copies/g of fresh matter (FM) sample, compared to a non-template control dissociation curve. Real-time PCR was performed with the ABI 7900 HT Sequence Detection System (PE Biosystems) using optical-grade 96-well plates.

Ammonia concentration in digestive samples was determined with the aid of a gas-sensitive electrode (Hach Co., Loveland, CO, United States) combined with a digital voltmeter (Crison GLP 22, Crison Instruments, S.A.; Barcelona, Spain). Three grams of acidified content were diluted (1:2) with 0.16 M NaOH, after homogenization samples were centrifuged (1500 × *g*) for 10 min. The ammonia released was measured in the supernatants as different voltages in mV according to a procedure previously described in Hermes et al. (2009) that was adapted from Diebold et al. (2004). The SCFA and lactic acid analyses were performed by gas chromatography. The samples were submitted to an acid-base treatment followed by an ether extraction and derivatization with *N*-(tert-butyldimethylsilyl)-*N*-methyl-trifluoroacetamide (MBTSTFA) plus 1% tert-butyldimethylchlorosilane (TBDMCS) agent, using the method of Richardson et al. (1989), modified by Jensen et al. (1995). For *Salmonella* bacteria counts, all samples were transferred (1:10) to BPW. Quantitative assessment was made by seeding the 10^-2^, 10^-4^ and 10^-6^ serial dilutions of the samples in Xylose-Lactose-Tergitol-4 (XLT4) plates (Merck; Madrid, Spain). The qualitative assessment was made by incubating samples in BPW (37°C, 24 h), transferring them to Rappaport-Vassiliadis enrichment broth (Oxoid; Hampshire, United Kingdom) for a second incubation (42°C, 48 h), and finally seeding them in XLT4 plates in order to observe H_2_S-positive colonies.

Tissue samples for morphological measures were dehydrated and embedded in paraffin wax, sectioned to a 4 μm thickness and stained with hematoxylin and eosin. Measurements of 10 different villous-crypt complexes per sample were performed with a light microscope (BHS, Olympus; Barcelona, Spain) using the technique described in Nofrarías et al. (2006).

Serum concentrations of Tumor Necrosis Factor-α (TNF-α) were determined by Quantikine Porcine TNF-α kits (R&D Systems; Minneapolis, MN, United States) according to the manufacturer’s instructions. Pig major acute-phase protein (Pig-MAP) concentrations were determined by a sandwich-type ELISA (Pig MAP Kit ELISA, Pig CHAMP Pro Europe S.A.; Segovia, Spain) as described in Saco et al. (2011). Serological antibodies of *Salmonella* were tested by ELISA *Salmonella* Herdcheck (Idexx; Hoofddorp, Netherlands), and the cut-off for positivity was established at optic density ≥40%.

### Statistical Analysis

Results are expressed as LS-Means with their standard errors unless otherwise stated. A two-way ANOVA was used to examine the effect of experimental challenge and probiotic treatment, as well as the interaction between the two (only included when significant). The general linear and mixed models of SAS (SAS Institute Inc., 2009) were used to analyze the effect of experimental treatments. For microbiological data, Fisher’s exact test was used to analyze the frequencies of positive animals as contingency tables, and the odds ratio (OR) with its 95% confidence interval was calculated on the basis of fixed effects.

When treatment effects were established, treatment means were separated using the probability-of-differences function adjusted by Tukey–Kramer. The pen was considered the experimental unit for analysis, and random effect was used to account for variation between pens. The α-level used for the determination of significance for all of the analysis was *P* = 0.05. The statistical trend was also considered for *P* < 0.10.

## Results

In general, the trial proceeded as expected. Animals showed a good health status at the beginning of the experiment. None of the animals seeded *Salmonella* in feces on arrival, and serological analysis confirmed that animals had not been exposed to *Salmonella* previous to the day of inoculation, all animals being analyzed as seronegative along the whole trial. During the PI period, three deaths and an euthanasia for ethical reasons were registered in the challenged groups; one from the CN group the 4th day after inoculation and three from the CP group on Days 3, 5, and 6 PI, all from different pens. Necropsy was performed on the dead animals. All of them presented fibrinohemorrhagic gastritis and acute diffuse fibrinous enteric-tiflo-colitis, lesions normally associated with infection of *Salmonella* Typhimurium in pigs (Wilcock and Olander, 1977). Although casualties in the CP group were more than those in the CN group (3/24 vs. 1/24), differences were not statistically significant. No antibiotic treatment was administered to any of the animals in the trial.

The ability of the probiotic strains to colonize the gut was indirectly evaluated by analyzing the total *Bifidobacterium* spp. copies in the colonic content on Day 8 PI. Mean concentrations of DNA copies/g of FM detected were 3.16 × 10^7^ for CN, 2.22 × 10^7^ for CP, 2.03 × 10^7^ for NN, and 8.11 × 10^7^ for NP. Probiotic and challenge effects were not significant, but a tendency was seen for the interaction challenge × probiotic (*P* = 0.058), where the number of DNA copies increased only in non-challenged animals receiving the probiotic.

### Animal Performance

Effects of the experimental treatments on BW, ADG, and ADFI are expressed in **Table 2**. The *Salmonella* challenge negatively affected final BW, ADFI, and ADG in the post-challenge period.

**Table 2:** Animal performance parameters.

	Treatments^a^				*P-*value			
	CN	CP	NN	NP	RSD^b^	*Challenge*	*Probiotic*	*Interaction*
**BW^c^ (kg)**					
Initial	7.90	7.51	7.60	7.85	0.559	0.929	0.770	0.195
Final	8.78	9.34	9.82	10.35	0.834	0.010	0.131	0.967
**ADFI^d^ (g/d)**					
Pre-inoculation^e^	259	306	273	250	55.9	0.391	0.611	0.164
Post-inoculation^f^	240	343	435	445	114.9	0.008	0.276	0.368
**ADG^g^ (g/d)**					
Pre-inoculation^e^	53	119	78	58	50.5	0.417	0.303	0.063
Post-inoculation^f^	13	97	273	300	122.5	<0.001	0.309	0.601

*^a^Treatments: CN, challenged + no probiotic; CP, challenged + probiotic; NN, no challenge + no probiotic; NP, no challenge + probiotic. ^b^Residual standard deviation. ^c^Body weight. ^d^Average daily feed intake. ^e^Experimental Days 0 to 7. ^f^Experimental days 8 to 16 (0 to 8 PI). ^g^Average Daily Gain. *n* = 8 for groups CN and CP, *n* = 4 for groups NN and NP.*

### Changes in Fermentative Activity

**Table 3** shows the changes promoted by the experimental treatments on the main ileal and colonic fermentation products.

**Table 3:** Colonic pH values, ammonia concentration and fermentation products for Days 4 and 8 post-inoculation (PI).

		Treatments^a^				*P*-value			
	Days PI	CN	CP	NN	NP	*RSD^b^*	*Challenge*	*Probiotic*	*Interaction*
**Ileum**					
pH	4	6.85	6.76	6.56	6.80	0.539	0.608	0.751	0.515
	8	6.83	6.76	6.66	6.28	0.409	0.099	0.239	0.419
NH3 *(mmol/L)*	4	4.47	2.37	2.95	5.13	1.77	0.455	0.961	0.016
	8	0.81	0.71	1.09	0.74	0.648	0.581	0.425	0.651
Acetic acid *(mmol/kg)*	4	2.58	5.06	3.03	3.95	4.280	0.887	0.403	0.706
	8	2.73	3.89	1.60	6.15	2.210	0.568	0.008	0.097
Lactic acid *(mmol/kg)*	4	19.8	37.0	54.4	58.6	34.74	0.096	0.515	0.691
	8	49.1	45.7	22.6	43.1	36.72	0.376	0.602	0.466
**Colon**					
pH	4	6.19	6.07	5.77	6.03	0.476	0.275	0.758	0.379
	8	6.14	5.96	5.82	6.11	0.254	0.473	0.626	0.058
NH3 *(mmol/L)*	4	6.59	5.71	4.89	5.62	4.398	0.643	0.967	0.678
	8	7.82	5.38	5.54	4.11	2.370	0.104	0.078	0.635
SCFA^c^ *(mmol/kg)*	4	98.4	94.1	136.2	138.5	39.42	0.030	0.957	0.853
	8	115.0	140.7	140.3	123.0	24.27	0.724	0.695	0.057
Lactic acid *(mmol/kg)*	4	1.00	4.82	5.10	8.43	5.644	0.141	0.170	0.923
	8	0.84	1.01	12.07	8.07	4.699	<0.001	0.363	0.324

*^a^Treatments: CN, challenged + no probiotic; CP, challenged + probiotic; NN, no challenge + no probiotic; NP, no challenge + probiotic. ^b^Residual Standard Deviation. *n* = 8 for groups CN and CP, *n* = 4 for groups NN and NP. ^c^SCFA include acetic, propionic butyric, valeric and branched-chain fatty acids.*

Probiotic treatment did not show significant effects on the studied parameters despite a tendency to interaction (*P* = 0.063) seen for the ADG before the challenge, the CP group showing a higher ADG than did its control (CN).

**FIGURE 1:**
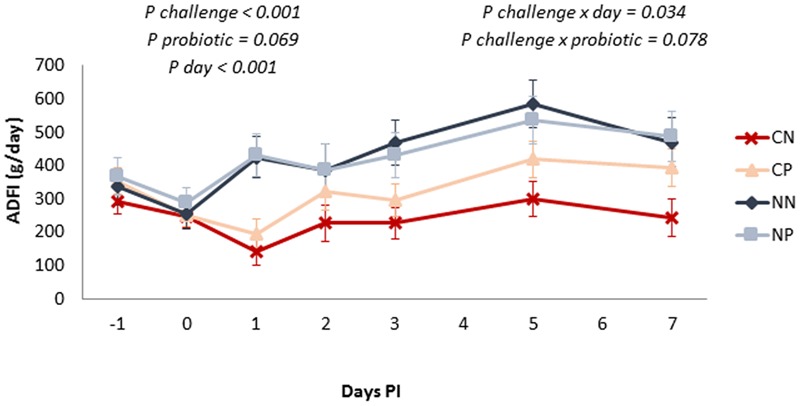
Average daily feed-intake evolution along the post-inoculation (PI) period. Treatments: CN, challenged + no probiotic; CP, challenged + probiotic; NN, no challenge + no probiotic; NP, no challenge + probiotic. *n* = 8 for groups CN and CP, *n* = 4 for groups NN and NP. Interactions only included when significant. Bars represent the standard error of the LS-Means.

Evolution of ADFI during the post-challenge period is shown in **Figure 1**. Feed intake was reduced by the *Salmonella* challenge (*P* < 0.001), this effect being especially manifested on Day 1 post-inoculation (interaction challenge × day *P* = 0.034). A tendency was found for the probiotic to enhance feed consumption (*P* = 0.069), and although not significantly, this effect was more manifested in the inoculated animals (interaction challenge × probiotic *P* = 0.078).

### Clinical Signs

**FIGURE 2:**
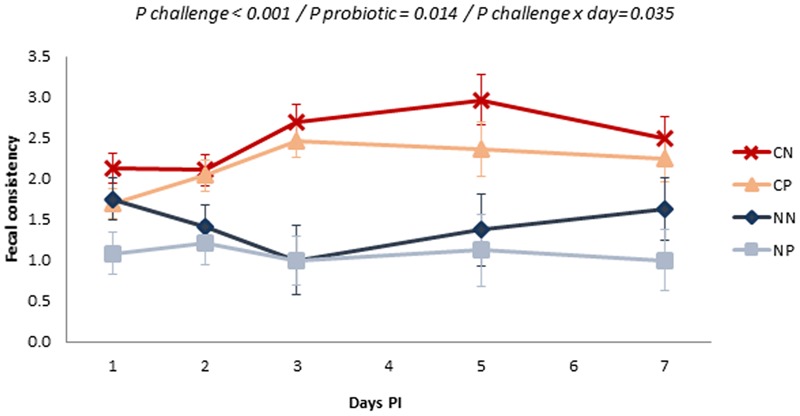
Evolution of the mean fecal scores in the different experimental groups along the post-inoculation (PI) period. Fecal score was measured using a scale from 1 (solid and cloddy) to 4 (watery or with blood). Treatments: CN, challenged + no probiotic; CP, challenged + probiotic; NN, no challenge + no probiotic; NP, no challenge + probiotic. *n* = 8 for groups CN and CP, *n* = 4 for groups NN and NP. *P*-values only included when significant. Bars represent the standard error of the LS-Means.

**Figure 2** shows the evolution of fecal consistency after the challenge. *Salmonella* inoculation significantly affected fecal scores with more liquid feces (*P* challenge < 0.001), especially from Day 3 onwards (*P* challenge × day = 0.035). Administration of the probiotic improved fecal consistency, with decreases in the fecal score in both challenged and non-challenged animals (*P* = 0.014).

On Day 1 PI, the challenged animals presented significantly higher rectal temperatures than did the non-challenged animals (40.0°C vs. 39.3°C, *P* < 0.001) despite the administration of the probiotic. However, on Day 2 PI, only the challenged animals not receiving the probiotic presented higher temperatures (39.9°C vs. 39.2°C, 39.1°C and 38.9°C for CN, CP, NN, and NP, respectively) (*P* interaction probiotic × day = 0.048).

### Salmonella Analysis

**FIGURE 3:**
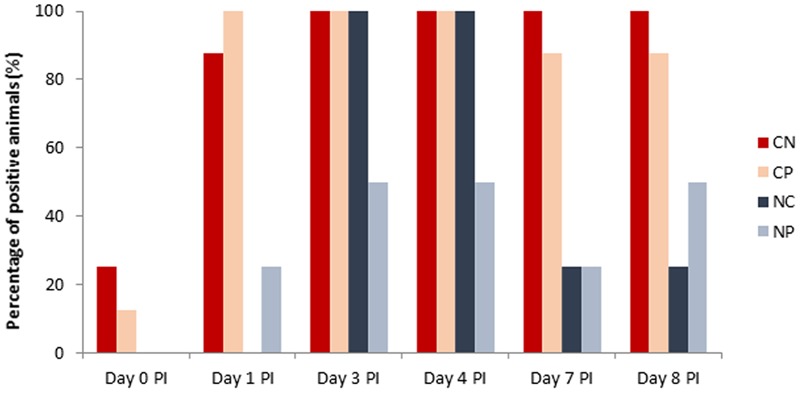
Percentage of positive (>1 cfu/g) animals to *Salmonella* spp. in feces (Day 0, Day 1, Days 3, and 7 post-inoculation [PI]) or colonic content (Days 4 and 8 PI). Treatments: CN, challenged + no probiotic; CP, challenged + probiotic; NN, no challenge + no probiotic; NP, no challenge + probiotic. *n* = 8 for groups CN and CP, *n* = 4 for groups NN and NP.

None of the analyzed animals seeded *Salmonella* on arrival. **Figure 3** shows the prevalence of positive animals to *Salmonella* during the post-challenge period. After the oral challenge with *Salmonella*, all animals except one that received the bacterial inoculum were positive in feces and stayed positive for all of the remaining experimental period. Unexpectedly, three animals were positive for *Salmonella* before inoculation (Day 0 PI), and some additional animals of the non-challenged group also became positive for *Salmonella* on Days 1, 3, 4, 7, and 8 PI. However, from all samples analyzed in the PI period, 98.7% of the samples of challenged animals were found to be positive (1–10^2^ cfu/g) during the PI period, while only 45% of the samples of non-challenged animals were positive. No significant effects were seen with the probiotic.

**FIGURE 4:**
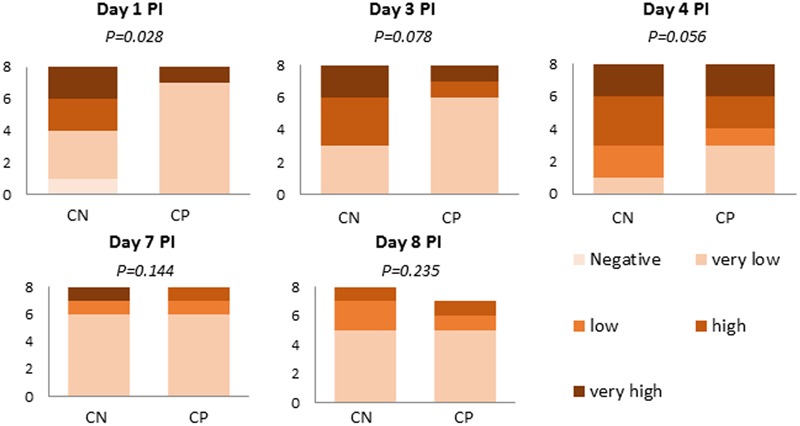
Number of animals in the different range levels of *Salmonella* spp. in feces (Days 1, 3, and 7 post-inoculation [PI]) or colonic digesta (Days 4 and 8 PI). Range Levels: Negative (0 cfu/g), Very low (1–10^2^ cfu/g), Low (10^3^–10^4^ cfu/g), High (10^5^–10^6^ cfu/g) and Very high (10^7^–10^8^ cfu/g). CN (challenged + no probiotic) and CP (challenged + probiotic). *n* = 8 for groups CN and CP (except *n* = 7 for CP on Day 8 PI).

**Figure 4** represents the semi-quantitative analysis of *Salmonella* in feces and colon digesta of those animals that received the pathogen inoculum. None of the non-challenged animals excreted *Salmonella* in quantifiable levels (>10^2^ cfu/g) and, therefore, they are not represented in the figure. The probiotic administration significantly lowered the number of animals with high *Salmonella* excretion levels on Day 1 (*P* = 0.028) and also tended to lower them on Days 3 (*P* = 0.078) and 4 (*P* = 0.056), with an increase in the frequency of the animals with less than 10^3^ cfu/g.

In the ileum, the *Salmonella* challenge caused a mild affection with a tendency to decrease ileal, lactic acid concentrations on Day 4 PI (*P* = 0.096) and to increase pH on Day 8 PI (*P* = 0.099). The colon was more severely affected, with a significant (*P* < 0.001) decrease in lactic acid concentrations on Day 8 PI and numerical decreases on Day 4 PI (*P* = 0.141). Significant decreases of colonic SCFA were also observed on Day 4 PI (*P* = 0.030) together with a tendency to increase ammonia concentrations (*P* = 0.104) on Day 8 PI.

Some beneficial changes were observed in the fermentation profile with the probiotic treatment. A significant increase in ileal acetic acid (*P* = 0.008) was seen on Day 8 PI, this increase being of a bigger magnitude in non-challenged animals (*P* challenge × probiotic = 0.097). Moreover, a tendency to decrease colonic ammonia concentrations (*P* = 0.078) was detected on Day 8 PI. Some changes promoted by the probiotic were not the same in challenged and non-challenged animals. Surprisingly, increased ileal ammonia levels were detected in the NP group, while a decrease was observed in CP animals in comparison to their control (*P* challenge × probiotic = 0.016). A trend for a similar pattern was observed for pH in the colon on Day 8 PI (*P* challenge × probiotic = 0.058). On the other hand, a tendency to decrease colonic total SCFA in the NP group and increase them in the CP group was seen on Day 8 PI (*P* challenge × probiotic = 0.057). Molar ratios of colonic SCFA were not significantly modified by any of the treatments; mean molar ratios detected in the trial were 62.7% acetic, 23.6% propionic, 9.8% butyric, 3.0% valeric and 1.1% branched-chain fatty acids.

### Immune Response

**Table 4** reports the serological concentrations of acute-phase proteins Pig-MAP and the pro-inflammatory cytokine TNF-α. The *Salmonella* challenge caused significant (*P* < 0.05) increases in both indexes, except on Pig-Map, on Day 8 PI, where the increase was numeric (*P* = 0.140). No significant effects were observed with the probiotic treatment although a numeric decrease of TNF-α was observed on Day 8 PI (*P* = 0.121) in animals receiving the probiotic treatment.

**Table 4:** Effects on serum levels of pro-inflammatory cytokine TNF-α and acute-phase protein Pig-MAP on Days 4 and 8 post-inoculation (PI).

	Treatments^a^				*P*-value			
	CN	CP	NN	NP	*RSD^b^*	*Challenge*	*Probiotic*	*Interaction*
**Pig-Map (mg/ml)**					
Day 4 PI	3.38	2.31	1.03	1.10	1.657	0.024	0.504	0.446
Day 8 PI	2.31	1.57	1.45	1.05	1.019	0.140	0.215	0.708
**TNF-α (pg/ml)**					
Day 4 PI	151	158	87.7	77.6	41.30	<0.001	0.927	0.647
Day 8 PI	112	82.6	63.9	61.6	21.95	0.002	0.121	0.182

*^a^Treatments: CN, challenged + no probiotic; CP, challenged + probiotic; NN, no challenge + no probiotic; NP, no challenge + probiotic. ^b^Residual Standard Deviation. *n* = 8 for groups CN and CP, *n* = 4 for groups NN and NP.*

### Intestinal Morphology

The histomorphological results of the ileum are summarized in **Table 5**. The challenge with *Salmonella* caused important decreases in villous height (*P* = 0.026 on Day 4; *P* = 0.061 on Day 8 PI), and although the crypt depth was not affected, the challenge significantly altered the villus:crypt ratio both days (*P* < 0.05). Additionally, an increase was seen in the number of goblet cells (GC) on Day 8 PI (*P* = 0.018) and on the number of mitosis on Day 4 PI (*P* = 0.022).

**Table 5:** Ileal histomorphometry on Days 4 and 8 post-inoculation (PI).

		Treatments^a^				*P*-value			
	Days PI	CN	CP	NN	NP	*RSD^b^*	*Challenge*	*Probiotic*	*Interaction*
Villous height (μm)	4	192.0	183.6	258.1	256.9	65.94	0.026	0.869	0.903
	8	198.3^B^	243.4^A,B^	303.3^A^	237.5^A,B^	56.80	0.061	0.682	0.038
Crypt depth (μm)	4	253.9	249.2	225.6	268.1	27.24	0.696	0.125	0.059
	8	242.5^A^	242.7^A^	182.8^B^	259.1^A^	30.70	0.124	0.010	0.011
Villous:Crypt ratio	4	0.78	0.75	1.15	0.96	0.280	0.027	0.382	0.509
	8	0.81^B^	1.01^B^	1.67^A^	0.93^B^	0.218	<0.001	0.011	<0.001
IEL^c^ (N° cel/100 μm)	4	1.04	1.25	1.38	1.49	0.546	0.243	0.506	0.822
	8	0.73	1.21	0.98	1.44	0.398	0.178	0.015	0.950
GC^d^ (N° cel/100 μm)	4	0.74	0.69	0.71	0.66	0.308	0.817	0.719	0.965
	8	0.96	1.04	0.66	0.44	0.401	0.018	0.697	0.402
Mitosis^e^ (N° cel/100 μm)	4	0.42	0.36	0.26	0.26	0.123	0.022	0.579	0.579
	8	0.25	0.26	0.34	0.20	0.097	0.765	0.150	0.103

*^a^Treatments: CN, challenged + no probiotic; CP, challenged + probiotic; NN, no challenge + no probiotic; NP, no challenge + probiotic. ^b^Residual Standard Deviation. *n* = 8 for groups CN and CP, *n* = 4 for groups NN and NP. **^c^**IEL = Villous intraepithelial lymphocytes; ^d^GC = Villous goblet cells/100 μm; ^e^Number of mitosis in crypts. ^AB^Means within rows without common letters differ by the Means-Tukey adjustment test (*P* < 0.05).*

Probiotic administration promoted a different effect on challenged and non-challenged animals. Whereas the probiotic moderately increased villous height in the challenged animals, it decreased it in the non-challenged ones (Day 8 PI; *P* challenge × probiotic = 0.038). Regarding crypts, the probiotic increased the crypt depth of the non-challenged animals until levels similar to the challenged ones (Day 8 PI; *P* challenge × probiotic = 0.011). These changes were reflected in the villous:crypt ratio (Day 8 PI; *P* challenge × probiotic < 0.001). Intraepithelial lymphocytes (IEL) were significantly increased by the probiotic on Day 8 PI in both groups (*P* = 0.015).

## Discussion

The aim of this study is to determine if the administration of the probiotic combination of *B. infantis* IM1^®^ and *B. lactis* BPL6 conferred protection against *Salmonella* Typhimurium.

A 2-week trial using weanling piglets was performed, where animals were challenged with *Salmonella* after 7 days of adaptation to the treatments and new environment. The challenge promoted an acute episode of diarrhea with increased fecal scores, fever response and the death (natural or euthanasia) of four animals that presented fibrinous-hemorrhagic lesions, normally associated with *Salmonella* Typhimurium (Wilcock and Olander, 1977). Virtually all parameters studied responded significantly to the pathogen inoculation: performance parameters, fermentation products, inflammatory response and ileal histomorphology were severely altered in comparison to non-challenged controls.

The administration of the probiotic combination was not able to prevent the infection of the animals by *Salmonella*, as mostly all of them became positive in feces 1 day after the challenge. Despite this, the probiotic was able to reduce the pathogen load in colon and feces, suggesting the potential of the bifidobacteria combination to exclude *Salmonella*. In this sense, it is also interesting to comment that, in our study, we unexpectedly found some animals becoming positive to *Salmonella* in feces in the non-challenged groups during the PI period, despite the seeding levels being very low (<10^2^ cfu/g). This can be attributed to some failure in the biosecurity protocol, as low concentrations of *Salmonella* in the environment (10^2^ to 10^3^ cfu) have been reported as being able to infect exposed animals (Hurd et al., 2001; Boughton et al., 2007). The fact that all fecal samples were negative for *Salmonella* on their arrival, and that all euthanized piglets remained seronegative at the end of the study, reaffirms that these animals were not previously exposed to the pathogen in the farm of origin (Nielsen et al., 1995). In those animals, it is interesting to point out that although all NN animals were positive in 2 days PI, the maximum percentage of positive animals for NP was 50% during all of the PI week. This could suggest that, although the probiotic was not able to prevent the infection of the animals when they were exposed to a high oral load of *Salmonella*, it could have some effect in front of a low exposure maintained over time. However, protection in our experiment was not significant, probably due to the low number of replicates in non-challenged animals (*n* = 4).

*In vitro* studies have shown the ability of bifidobacteria to inhibit the growth of *Salmonella* (Bielecka et al., 1998; Tanner et al., 2016). A pluripotent stimulatory effect on the immune system (Gill et al., 2001; Medina et al., 2007; Akahashi et al., 2013), production of organic acids (Saulnier et al., 2009), production of bacteriocins and bacteriocin-like substances (Cheikhyoussef et al., 2008), and the capacity to inhibit the pathogenic adhesion to enterocytes or prevent bacterial translocation (Gagnon et al., 2004; Searle et al., 2009) have been described as the possible mechanisms of action of *Bifidobacterium* spp. for these antimicrobial effects. Regarding the strains evaluated in this study, previous works using a similar model of disease have also demonstrated the potential of the *B. infantis* IM1^®^ strain to reduce *Salmonella* loads (Barba-Vidal et al., 2017). For this *B. infantis* IM1^®^ strain, several mechanisms have been reported that could be involved in a favorable outcome: the potential to produce peptides with protease activity (Chenoll et al., 2016), immunomodulatory capacity by increasing IL-10 (unpublished data) and IgA (Moreno Muñoz et al., 2011) production have been reported.

In accordance with reductions in the pathogen loads, improvements in clinical parameters were also registered with the probiotic combination. Rectal temperature of the CP group returned to levels similar to those of the non-challenged groups 48 h after the pathogen inoculation, and fecal scores showed less diarrhea. Regarding diarrhea scores, it is also interesting to remark that they were reduced not only in the CP group but also in the NP group. After weaning, piglets suffer stress for several abrupt changes: separation from the sow, change from a milk-based to a less-digestible dry-cereal-based feed diet, introduction to new social partners and new physical environments (Weary et al., 2008), which usually promote gut dysbiosis. Our results suggest that the probiotic treatment may not only improve piglet outcome against pathogens, but it may also help piglets in a post-weaning period.

Differences in productive parameters are rarely reported in challenge trials evaluating probiotic treatments, as these studies are usually run in short periods and with a limited number of animals. Despite this, we were able to see trends for a positive effect of the probiotic in the intake of feed during the post-challenge period, more manifested in the CP group, and also a numerically higher final live-weight (*P* = 0.131) in the animals receiving the probiotic, with more than 500 g of difference at the end of the study. In this study, this increase in feed intake and weight should be considered as a sign of better health status and adaptation to the weaning stress that allows the animal to express its genetic growth potential. These positive results should not be considered as a risk for obesity, as has clearly been stated by Bernardeau and Vernoux (2013) for the extrapolation of farm-animal results to humans.

Modulation of the fermentation profile was also detected with the probiotic treatment at the ileal and colonic level. A general increase in ileal acetic acid concentrations (more importantly in the NP group) was registered on Day 8 PI. Scientific literature reports that carbohydrate degradation by bifidobacteria exclusively takes place by the characteristic fructose-6-phosphate shunt (or bifidus pathway). Acetic acid and lactic acid are the major end-metabolites (de Vries and Stouthamer, 1967; Van der Meulen et al., 2006), with a theoretical molar ratio of acetic acid to lactic acid of 1.5 (de Vries and Stouthamer, 1968; Van der Meulen et al., 2006). Considering this, we speculate that increases in acetic acid could be due to a higher bifidobacterial presence in the ileum in animals treated with probiotic although we cannot discard other bacterial species to be involved too. Lactic acid, as the main product of most of the inhabitants of the small intestine (Clemens and Stevens, 1979), would not have been sensitive enough to reflect changes. This increase in acetic acid was more manifested in non-challenged animals on Day 8 PI, reflecting a more established ileal microbiota at this time. Actually, on Day 8 PI, we were also able to detect a higher number of *Bifidobacterium* spp. in the colon by qPCR quantification in the non-challenged animals receiving the probiotic. However, changes in ileal fermentation were not always favorable. An interaction effect increasing ileal ammonia concentrations in NP and decreasing in CP was observed on Day 4 PI. We cannot find an explanation for this; however, it is worth mentioning that this effect disappeared on Day 8 PI with a substantial reduction in ammonia mean values for all treatments. This evolution in ileal ammonia could be a reflection of the big transitions of microbial populations that are produced during first days after weaning (Wang et al., 2013), regardless of the experimental treatment the animals received.

In relation to colonic fermentation, some benefits related to the probiotic were observed on Day 8 PI. Firstly, a reduction in ammonia concentration was reported (*P* = 0.078). This effect could be due to the ability to utilize ammonia attributed to bifidobacteria or an indirect effect via modulation of the fermentation profile (Arunachalam, 1999; Ahasan et al., 2015) with a reduction in the proteolytic populations. Secondly, an interaction in SCFA on Day 8 PI was observed. The concentration increased in CP groups to levels similar to those of the NN group and could suggest a normalization of the fermentative activity with the probiotic in challenged animals. Nevertheless, SCFA were decreased by the probiotic in the non-challenged group. This reduction would be generally accepted as a negative impact of the probiotic on gut health. However, in our case it can also be a natural consequence of a reduced amount of fermentable substrates arriving in the colon. The abrupt increase observed for acetic acid in the ileum of these animals could suggest a more active microbial population at the end of the small intestine that could have reduced the amount of substrates susceptible to be fermented in the colon. Differentially to humans, the fermentative activity at the end of the small intestine in pigs is quite important.

Interesting results were also observed for ileal histomorphometry on Day 8 PI. Whereas the probiotic treatment maintained villous height of challenged animals to similar levels as of non-challenged, numerical reductions in the NP group were observed. Moreover, in the non-challenged animals the probiotic also promoted an increase in crypt depths up to similar values of the challenged animals. Subsequently, changes were reflected accordingly in the villous:crypt ratio. These results could again be related to a higher colonization of the ileum by the probiotic bacteria and to the presence of a greater ileal fermentative activity in these animals. In consonance, Kleessen et al. (2003) observed increased jejunal villus height and crypt depth in a rat model of human fecal microbiota due to fermentation of fructans by bifidobacteria. It is also important to keep the great capacity to adhere to enterocytes reported for bifidobacteria in mind (Servin, 2003; Collado et al., 2005, Collado et al., 2007a), which has been reported to be increased in combination of probiotic strains including *Bifidobacterium* spp. (Collado et al., 2007a). We suspect that the presence of highly adhesive bifidobacteria could have contributed to the exclusion of intestinal pathogens (Collado et al., 2005) in challenged animals, as the CN group had a more severely affected villous height. However, the adhesion of these bifidobacteria in the non-challenged animals, and the increased fermentative activity observed in the ileum, could have somehow compromised villous enterocytes in the NP group. To our knowledge, it is the first time that such a reduction on villous height has been described due to a bifidobacterial probiotic. Still, similar results were also found for this probiotic combination by our group in other non-published studies, proving that these effects are quite consistent. Nevertheless, none of the parameters analyzed in this study suggests negative effects of the probiotic in this group, and TNF-α showed the lowest values in them, suggesting no deleterious effects on the intestine. Interestingly, IEL mean values were decreased between Days 4 and 8 PI only in the animals not receiving the probiotic, despite their being challenged or not with *Salmonella*. Possibly the increased values observed on Day 4 were due to opportunistic pathogens taking advantage of a transient dysbiosis related to weaning. On Day 8, only the animals receiving the bifidobacteria maintained those response levels, suggesting the ability of the probiotic strains to stimulate the immune system of the animal.

Bifidobacteria are considered to be minor colonizers of swine gut post-weaning (Konstantinov et al., 2004; Zhao et al., 2015). Colonic concentrations of *Bifidobacterium* spp. reported in the piglet colon range from 10^5^ to 10^8^ cfu/g (Mountzouris et al., 2006; Fouhse et al., 2015), and Mountzouris et al. (2006) estimated that they contribute to approximately 0.4 ± 0.15% of total bacteria in the ascending colon. In our study, total *Bifidobacterium* spp. was analyzed via qPCR and, despite a 10^9^ daily dose of combined *Bifidobacteria* being used, noticeable increases were only observed in the non-challenged animals. Low detection levels in the challenge animals could respond to the gut dysbiosis produced by the *Salmonella* challenge that had precluded the probiotic to fully colonize the gut. Furthermore, it should be considered that at the moment the samples were taken, the animals had not received the probiotic in the previous 24 h. Therefore, considering that transit time is accelerated in intestinal disorders, unless the probiotic strains had colonized the gut, it would have been very improbable to detect it in the colonic samples. The lack of response of the qPCR numbers for *Bifidobacterium* spp. also could respond to a substitution between species, maintaining the niche a similar size. For this reason, although we cannot demonstrate the colonization of the gut by the probiotic strains, it cannot be discarded either. Even in the eventual case of the strains not colonizing the gut, another possible explanation for the effects observed with the probiotic could be that effects were mediated by metabolic products or other bioactive compounds and not by the bacteria cells themselves. In this regard *Bifidobacterium infantis* immunomodulation seems to be at least partially regulated by bioactive peptides which can retain their biological activity even without the strain being present (Ewaschuk et al., 2008). In this line, a recent publication demonstrates that one of the strains included in this combination, *B. infantis* IM1^®^, produces peptides with protease activity (Chenoll et al., 2016).

As it could be seen, the probiotic combination evaluated in this study has demonstrated a clear positive effect, not only ameliorating the *Salmonella* challenge outcome but also improving weaning response. These results are better than previous ones obtained by our group for the *B. infantis* IM1^®^ single strain (Barba-Vidal et al., 2017) with a similar experimental design. In that study, the use of the *B. infantis* IM1^®^ strain also diminished *Salmonella* shedding, although challenged animals treated with the probiotic failed to show the significant improvements observed in this study in clinical outcomes, fermentation or histomorphometry. Other authors have also described the benefits of using a combination of different strains (Perdigon et al., 1990; Casey et al., 2007). However, unfortunately, *in vivo* studies comparing the effect of single strains with the strain combination are still rare (Chapman et al., 2011). In our study, the reported results suggest that the evaluated combination of bifidobacterial strains showed a better outcome that results previously reported for the single strain *B. infantis* IM1^®^ (Barba-Vidal et al., 2017). However, our experimental design does not allow us to identify if the reason for this improvement is due complementary effects of the strains, as both strains were not tested individually in this study.

## Conclusion

The probiotic combination of *B. infantis* IM1^®^ and *B. lactis* BPL6 had a positive effect on enhancing gut health on post-weaning piglets and alleviating animals in a *Salmonella* challenge. Improvements registered in challenged animals were a reduction of the fecal excretion of *Salmonella* Typhimurium, a decrease in rectal temperature to similar levels to that in non-challenged animals and improvements in the villous:crypt ratio. In addition, general probiotic benefits were observed in both challenged and non-challenged groups, showing an increase in voluntary feed-intake, a decrease of diarrhea scores, healthier fermentation profiles and a stimulation of the intestinal immune system by increasing IEL.

## Author Contributions

EB-V participated in the experimental design, was responsible for the animal trial, laboratory analysis, data analysis, and writing. LC participated in the experimental design, animal trials, data analysis and writing. VR participated in animal trials, data analysis, and writing. GC-O and JM participated in the experimental design, contributed to data analysis, and writing. SM-O participated in the experimental design, animal trials, laboratory analysis, data analysis, and writing.

## Conflict of Interest Statement

This work was partially funded by Laboratorios Ordesa. GC-O and JM are employees of Laboratorios Ordesa S.L. The others authors declare that the research was conducted in the absence of any commercial or financial relationships that could be construed as a potential conflict of interest

## References

[B1] Ahasan A. Agazzi A. Invernizzi G. Bontempo V. Savoini G. (2015). The beneficial role of probiotics in monogastric animal nutrition and health. *J. Dairy Vet. Anim. Res.* 2 1–20. 10.15406/jdvar.2015.2.00041

[B2] Akahashi N. T. Itazawa H. K. Wabuchi N. I. Iao J. X. Iyaji K. M. Watsuki K. I. (2013). Oral administration of an immunostimulatory DNA sequence from *Bifidobacterium longum* improves Th1/Th2 balance in a murine model. *Biosci. Biotechnol. Biochem.* 70 2013–2017. 10.1271/bbb.60260 16926520

[B3] AOAC International (1995). *Official Methods of Analysis of AOAC International.* Arlington, VA: AOAC International.

[B4] Arunachalam K. D. (1999). Role of Bifidobacteria in nutrition, medicine and technology. *Nutr. Res.* 19 1559–1597. 10.1016/S0271-5317(99)00112-8

[B5] Barba-Vidal E. Castillejos L. López Colom P. Rivero Urgell M. Muñoz J. A. M. Martín Orúe S. M. (2017). Evaluation of the probiotic strain *Bifidobacterium longum* subsp. *infantis* CECT 7210 capacities to improve health status and fight digestive pathogens in a piglet model. *Front. Microbiol.* 8:533 10.3389/FMICB.2017.00533 PMC5386966 28443068

[B6] Bernardeau M. Vernoux J.-P. (2013). Overview of differences between microbial feed additives and probiotics for food regarding regulation, growth promotion effects and health properties and consequences for extrapolation of farm animal results to humans. *Clin. Microbiol. Infect.* 19 321–330. 10.1111/1469-0691.12130 23445377

[B7] Bielecka M. Biedrzycka E. Biedrzycka E. Smoragiewicz W. Smieszek M. (1998). Interaction of *Bifidobacterium* and *Salmonella* during associated growth. *Int. J. Food Microbiol.* 45 151–155. 10.1016/S0168-1605(98)00150-0 9924946

[B8] Bin-Nun A. Bromiker R. Wilschanski M. Kaplan M. Rudensky B. Caplan M. (2005). Oral probiotics prevent necrotizing enterocolitis in very low birth weight neonates. *J. Pediatr.* 147 192–196. 10.1016/j.jpeds.2005.03.054 16126048

[B9] Boughton C. Egan J. Kelly G. Markey B. Leonard N. (2007). Rapid infection of pigs following exposure to environments contaminated with different levels of *Salmonella typhimurium*. *Foodborne Pathog. Dis.* 4 33–40. 10.1089/fpd.2006.58 17378706

[B10] Casey P. G. Gardiner G. E. Casey G. Bradshaw B. Lawlor P. G. Lynch P. B. (2007). A five-strain probiotic combination reduces pathogen shedding and alleviates disease signs in pigs challenged with *Salmonella enterica* serovar typhimurium. *Appl. Environ. Microbiol.* 73 1858–1863. 10.1128/AEM.01840-06 17261517 PMC1828830

[B11] CDC (2013). *National Salmonella Surveillance Annual Report, 2012.* Atlanta, GA: CDC.

[B12] Chapman C. M. C. Gibson G. R. Rowland I. (2011). Health benefits of probiotics: Are mixtures more effective than single strains? *Eur. J. Nutr.* 50 1–17. 10.1007/s00394-010-0166-z 21229254

[B13] Cheikhyoussef A. Pogori N. Chen W. Zhang H. (2008). Antimicrobial proteinaceous compounds obtained from bifidobacteria: from production to their application. *Int. J. Food Microbiol.* 125 215–222. 10.1016/j.ijfoodmicro.2008.03.012 18514343

[B14] Chenoll E. Casinos B. Bataller E. Buesa J. Ramón D. Genovés S. (2016). Identification of a peptide produced by *Bifidobacterium longum* CECT 7210 with antirotaviral activity. *Front. Microbiol.* 7:655 10.3389/fmicb.2016.00655 PMC4855034 27199974

[B15] Clemens E. T. Stevens C. E. (1979). Sites of organic acid production and patterns of digesta movement in the gastro-intestinal tract of the raccoon. *J. Nutr.* 109 1110–1116. 448450 10.1093/jn/109.6.1110

[B16] Collado M. Meriluoto J. Salminen S. (2007c). Role of commercial probiotic strains against human pathogen adhesion to intestinal mucus. *Lett. Appl. Microbiol.* 45 454–460. 10.1111/j.1472-765X.2007.02212.x 17897389

[B17] Collado M. C. Grześkowiak Ł. Salminen S. (2007a). Probiotic strains and their combination inhibit *in vitro* adhesion of pathogens to pig intestinal mucosa. *Curr. Microbiol.* 55 260–265. 10.1007/s00284-007-0144-8 17657533

[B18] Collado M. C. Gueimonde M. Hernández M. Sanz Y. Salminen S. (2005). Adhesion of selected *Bifidobacterium* strains to human intestinal mucus and the role of adhesion in enteropathogen exclusion. *J. Food Prot.* 68 2672–2678. 10.4315/0362-028X-68.12.2672 16355841

[B19] Collado M. C. Meriluoto J. Salminen S. (2007b). Development of new probiotics by strain combinations: Is it possible to improve the adhesion to intestinal mucus? *J. Dairy Sci.* 90 2710–2716. 10.3168/jds.2006-456 17517710

[B20] de Vries W. Stouthamer A. H. (1967). Pathway of glucose fermentation in relation to the taxonomy of bifidobacteria. *J. Bacteriol.* 93 574–576. 6020562 10.1128/jb.93.2.574-576.1967 PMC276478

[B21] de Vries W. Stouthamer A. H. (1968). Fermentation of glucose, lactose, galactose, mannitol, and xylose by bifidobacteria. *J. Bacteriol.* 96 472–478. 5674058 10.1128/jb.96.2.472-478.1968 PMC252320

[B22] Diebold G. Mosenthin R. Piepho H.-P. Sauer W. C. (2004). Effect of supplementation of xylanase and phospholipase to a wheat-based diet for weanling pigs on nutrient digestibility and concentrations of microbial metabolites in ileal digesta and feces. *J. Anim. Sci.* 82 2647–2656. 10.2527/2004.8292647x 15446482

[B23] EFSA (2013). The European Union summary report on trends and sources of zoonoses, zoonotic agents and food-borne outbreaks in 2011. *EFSA J.* 11:3129 10.2903/j.efsa.2013.3129 PMC7009540 32625785

[B24] European Commission (2010). Directive 2010/63/EU of the European Parliament and of the Council of 22 September 2010 on the protection of animals used for scientific purposes. *Off. J. Eur. Union* 53 L276:33–52.

[B25] Ewaschuk J. B. Diaz H. Meddings L. Diederichs B. Dmytrash A. Backer J. (2008). Secreted bioactive factors from *Bifidobacterium infantis* enhance epithelial cell barrier function. *Am. J. Physiol. Gastrointest. Liver Physiol.* 295 G1025–G1034. 10.1152/ajpgi.90227.2008 18787064

[B26] Fouhse J. M. Gänzle M. G. Regmi P. R. van Kempen T. A. T. G. Zijlstra R. T. (2015). High amylose starch with low in vitro digestibility stimulates hindgut fermentation and has a bifidogenic effect in weaned pigs. *J. Nutr.* 145 2464–2470. 10.3945/jn.115.214353 26377761

[B27] Gagnon M. Kheadr E. E. Le Blay G. Fliss I. (2004). *In vitro* inhibition of *Escherichia coli* O157:H7 by bifidobacterial strains of human origin. *Int. J. Food Microbiol.* 92 69–78. 10.1016/j.ijfoodmicro.2003.07.010 15033269

[B28] Gill H. S. (2003). Probiotics to enhance anti-infective defences in the gastrointestinal tract. *Best Pract. Res. Clin. Gastroenterol.* 17 755–773. 10.1016/S1521-6918(03)00074-X 14507586

[B29] Gill H. S. Shu Q. Lin H. Rutherfurd K. J. Cross M. L. (2001). Protection against translocating *Salmonella* typhimurium infection in mice by feeding the immuno-enhancing probiotic *Lactobacillus rhamnosus* strain HN001. *Med. Microbiol. Immunol.* 190 97–104. 10.1007/s004300100095 11827205

[B30] Hermes R. G. Molist F. Ywazaki M. Nofrarías M. Gomez de Segura A. Gasa J. (2009). Effect of dietary level of protein and fiber on the productive performance and health status of piglets. *J. Anim. Sci.* 87 3569–3577. 10.2527/jas.2008-1241 19648494

[B31] Hurd H. S. Gailey J. K. McKean J. D. Rostagno M. H. (2001). Rapid infection in market-weight swine following exposure to a *Salmonella* Typhimurium-contaminated environment. *Am. J. Vet. Res.* 62 1194–1197. 10.2460/ajvr.2001.62.1194 11497437

[B32] Jensen M. T. Cox R. P. Jensen B. B. (1995). Microbial production of skatole in the hind gut of pigs given different diets and its relation to skatole deposition in backfat. *Anim. Sci.* 61 293–304. 10.1017/S1357729800013837

[B33] Kleessen B. Hartmann L. Blaut M. (2003). Fructans in the diet cause alterations of intestinal mucosal architecture, released mucins and mucosa-associated bifidobacteria in gnotobiotic rats. *Br. J. Nutr.* 89 597–606. 10.1079/BJN2002827 12720580

[B34] Konstantinov S. R. Favier C. F. Zhu W. Y. Williams B. A. Kl J. Souffrant W.-B. (2004). Microbial diversity studies of the porcine gastrointestinal ecosystem during weaning transition. *Anim. Res.* 53 317–324. 10.1051/animres:2004019

[B35] Lanata C. F. Fischer-Walker C. L. Olascoaga A. C. Torres C. X. Aryee M. J. Black R. E. (2013). Global causes of diarrheal disease mortality in children<5 years of age: a systematic review. *PLoS ONE* 8:e72788 10.1371/journal.pone.0072788 PMC3762858 24023773

[B36] Liévin V. Peiffer I. Hudault S. Rochat F. Brassart D. Neeser J. R. (2000). *Bifidobacterium* strains from resident infant human gastrointestinal microflora exert antimicrobial activity. *Gut* 47 646–652. 10.1136/gut.47.5.646 11034580 PMC1728100

[B37] Majowicz S. E. Musto J. Scallan E. Angulo F. J. Kirk M. O’Brien S. J. (2010). The global burden of nontyphoidal *Salmonella* gastroenteritis. *Clin. Infect. Dis.* 50 882–889. 10.1086/650733 20158401

[B38] Medina M. Izquierdo E. Ennahar S. Sanz Y. (2007). Differential immunomodulatory properties of *Bifidobacterium logum* strains: relevance to probiotic selection and clinical applications. *Clin. Exp. Immunol.* 150 531–538. 10.1111/j.1365-2249.2007.03522.x 17956582 PMC2219384

[B39] Minocha A. (2009). Probiotics for preventive health. *Nutr. Clin. Pract.* 24 227–241. 10.1177/0884533608331177 19321897

[B40] Moreno Muñoz J. A. Chenoll E. Bataller E. Ramón D. Genovés S. Montava R. (2011). Novel probiotic *Bifidobacterium longum* subsp. *infantis* CECT 7210 strain active against rotavirus. *Appl. Environ. Microbiol.* 77 8775–8783. 10.1128/AEM.05548-11 22003027 PMC3233071

[B41] Mountzouris K. C. Balaskas C. Fava F. Tuohy K. M. Gibson G. R. Fegeros K. (2006). Profiling of composition and metabolic activities of the colonic microflora of growing pigs fed diets supplemented with prebiotic oligosaccharides. *Anaerobe* 12 178–185. 10.1016/j.anaerobe.2006.04.001 16731014

[B42] Nielsen B. Baggesen D. Bager F. Haugegaard J. Lind P. (1995). The serological response to *Salmonella* serovars *typhimurium* and *infantis* in experimentally infected pigs. The time course followed with an indirect anti-LPS ELISA and bacteriological examinations. *Vet. Microbiol.* 47 205–218. 10.1016/0378-1135(95)00113-1 8748536

[B43] Nofrarías M. Manzanilla E. G. Pujols J. Gibert X. Majó N. Segalés J. (2006). Effects of spray-dried porcine plasma and plant extracts on intestinal morphology and on leukocyte cell subsets of weaned pigs. *J. Anim. Sci.* 84 2735–2742. 10.2527/jas.2005-414 16971575

[B44] NRC (2012). *Nutrient Requirements of Swine.* Washington, DC: National Academy Press.

[B45] Perdigon G. Nader de Macias M. E. Alvarez S. Oliver G. Pesce de Ruiz Holgado A. A. (1990). Prevention of gastrointestinal infection using immunobiological methods with milk fermented with *Lactobacillus casei* and *Lactobacillus acidophilus*. *J. Dairy Res.* 57 255–264. 10.1017/S002202990002687X 2111829

[B46] Richardson A. J. Calder A. G. Stewart C. S. Smith A. (1989). Simultaneous determination of volatile and non-volatile acidic fermentation products of anaerobes by capillary gas chromatography. *Lett. Appl. Microbiol.* 9 5–8. 10.1111/j.1472-765X.1989.tb00278.x

[B47] Saco Y. Fraile L. Giménez M. Alegre A. López-Jimenez R. Cortey M. (2011). Serum acute phase proteins as biomarkers of pleuritis and cranio-ventral pulmonary consolidation in slaughter-aged pigs. *Res. Vet. Sci.* 91 52–57. 10.1016/j.rvsc.2010.08.016 20932541

[B48] Salazar N. Ruas-Madiedo P. Kolida S. Collins M. Rastall R. Gibson G. (2009). Exopolysaccharides produced by *Bifidobacterium longum* IPLA E44 and *Bifidobacterium animalis* subsp. *lactis* IPLA R1 modify the composition and metabolic activity of human faecal microbiota in pH-controlled batch cultures. *Int. J. Food Microbiol.* 135 260–267. 10.1016/j.ijfoodmicro.2009.08.017 19735956

[B49] SAS Institute Inc. (2009). *SAS/STAT^®^9.2 User’s Guide* 2nd Edn. Cary, NC: SAS Institute Inc.

[B50] Saulnier D. M. A. Spinler J. K. Gibson G. R. Versalovic J. (2009). Mechanisms of probiosis and prebiosis: considerations for enhanced functional foods. *Curr. Opin. Biotechnol.* 20 135–141. 10.1016/j.copbio.2009.01.002 19243931 PMC2713183

[B51] Searle L. E. J. Best A. Nunez A. Salguero F. J. Johnson L. Weyer U. (2009). A mixture containing galactooligosaccharide, produced by the enzymic activity of *Bifidobacterium bifidum*, reduces *Salmonella enterica* serovar *Typhimurium* infection in mice. *J. Med. Microbiol.* 58 37–48. 10.1099/jmm.0.004390-0 19074651

[B52] Servin A. L. (2003). Adhesion of probiotic strains to the intestinal mucosa and interaction with pathogens. *Best Pract. Res. Clin. Gastroenterol.* 17 741–754. 10.1016/S1521-6918(03)00052-0 14507585

[B53] Shu Q. Lin H. Rutherfurd K. J. Fenwick S. G. Prasad J. Gopal P. K. (2000). Dietary *Bifidobacterium lactis* (HN019) enhances resistance to oral *Salmonella typhimurium* infection in mice. *Microbiol. Immunol.* 44 213–222. 10.1111/j.1348-0421.2000.tb02486.x 10832963

[B54] Silva A. M. Barbosa F. H. F. Duarte R. Vieira L. Q. Arantes R. M. E. Nicoli J. R. (2004). Effect of *Bifidobacterium longum* ingestion on experimental salmonellosis in mice. *J. Appl. Microbiol.* 97 29–37. 10.1111/j.1365-2672.2004.02265.x 15186439

[B55] Tanner S. A. Chassard C. Rigozzi E. Lacroix C. Stevens M. J. A. (2016). *Bifidobacterium thermophilum* RBL67 impacts on growth and virulence gene expression of *Salmonella enterica* subsp. *enterica* serovar Typhimurium. *BMC Microbiol.* 16:46 10.1186/s12866-016-0659-x PMC4797131 26988691

[B56] Thanabalasuriar A. Kubes P. (2014). Neonates, antibiotics and the microbiome. *Nat. Med.* 20 469–470. 10.1038/nm.3558 24804751

[B57] Timmerman H. M. Koning C. J. M. Mulder L. Rombouts F. M. Beynen A. C. (2004). Monostrain, multistrain and multispecies probiotics—a comparison of functionality and efficacy. *Int. J. Food Microbiol.* 96 219–233. 10.1016/j.ijfoodmicro.2004.05.012 15454313

[B58] Van der Meulen R. Adriany T. Verbrugghe K. De Vuyst L. (2006). Kinetic analysis of bifidobacterial metabolism reveals a minor role for succinic acid in the regeneration of NAD^+^ through its growth-associated production. *Appl. Environ. Microbiol.* 72 5204–5210. 10.1128/AEM.00146-06 16885266 PMC1538715

[B59] Van Soest P. J. Robertson J. B. Lewis B. A. (1991). Methods for dietary fiber, neutral detergent fiber, and nonstarch polysaccharides in relation to animal nutrition. *J. Dairy Sci.* 74 3583–3597. 10.3168/jds.S0022-0302(91)78551-2 1660498

[B60] Wang M. Radlowski E. C. Monaco M. H. Fahey G. C. Gaskins H. R. Donovan S. M. (2013). Mode of delivery and early nutrition modulate microbial colonization and fermentation products in neonatal piglets. *J. Nutr.* 143 795–803. 10.3945/jn.112.173096 23616518

[B61] Weary D. M. Jasper J. Hötzel M. J. (2008). Understanding weaning distress. *Appl. Anim. Behav. Sci.* 110 24–41. 10.1016/j.applanim.2007.03.025

[B62] Wilcock B. P. Olander H. J. (1977). The pathogenesis rectal structure. II. Experimental salmonellosis and ischemic proctitis. *Vet. Pathol.* 14 43–55. 10.1177/030098587701400106 850994

[B63] Zacarías M. F. Reinheimer J. Forzani L. Grangette C. Vinderola G. (2014). Mortality and translocation assay to study the protective capacity of *Bifidobacterium lactis* INL1 against *Salmonella* Typhimurium infection in mice. *Benef. Microbes* 5 427–436. 10.3920/BM2013.0086 24902954

[B64] Zhao W. Wang Y. Liu S. Huang J. Zhai Z. He C. (2015). The dynamic distribution of porcine microbiota across different ages and gastrointestinal tract segments. *PLoS ONE* 10:e0117441 10.1371/journal.pone.0117441 PMC4331431 25688558

